# P-1091. Hydrogen peroxide generating electrochemical bandage for the treatment of wound biofilm infections - preliminary demonstration of device safety on healthy human skin

**DOI:** 10.1093/ofid/ofae631.1279

**Published:** 2025-01-29

**Authors:** Paige Kies, Derek Fleming, Won-Jun Kim, Md Monzurul Anoy, Ibrahim Bozyel, Susan Holtegaard, Kerryl Greenwood-Quaintance, Haluk Beyenal, Robin Patel

**Affiliations:** Mayo Clinic, Rochester, Minnesota; Mayo Clinic, Rochester, Minnesota; Washington State University, Pullman, Washington; Washington State University, Pullman, Washington; Washington State University, Pullman, Washington; Mayo Clinic, Rochester, Minnesota; Mayo Clinic, Rochester, Minnesota; Washington State University, Pullman, Washington; Mayo Clinic, Rochester, Minnesota

## Abstract

**Background:**

Wound biofilm infections impact ∼2% of the United States and at-risk individuals often have underlying morbidities such as diabetes. Antibiotics and topical approaches like debridement or silver dressings have had limited success, underscoring a need for strategies that efficiently target pathogens in wound beds.

**Methods:**

We developed a hydrogen peroxide (H_2_O_2_) generating electrochemical bandage (e-bandage) composed of carbon fabric electrodes separated by cotton fabric with an Ag/AgCl reference electrode (RE) placed between cotton fabric layers. The e-bandage is controlled by a wearable micropotentiostat (MP); H_2_O_2_ is generated by O_2_ + 2H^+^+ 2e^-^ → H_2_O_2_. O_2_ is dissolved on a primary working electrode with negative polarity compared to the RE while a positively polarized secondary counter electrode generates H^+^ and e^-^. The H_2_O_2_ e-bandage/MP device has *in vitro* activity against mono- and dual-species bacterial and fungal biofilms. We showed that the H_2_O_2_ e-bandage decreased the burden of methicillin-resistant *Staphylococcus aureus* and/or *Pseudomonas aeruginosa* with difficult-to-treat resistance in a murine wound infection model. Given this, we initiated a H_2_O_2_ e-bandage safety assessment on healthy human skin. Here, testing of a non-polarized, circular (2 cm diameter) device pre-loaded with biocompatible hydrogel containing 0.9% NaCl is reported. Sterilized e-bandages assessed in a shelf-life study retained activity for two weeks. Adults with intact forearm skin were then recruited to the Clinical Research Trials Unit at the Mayo Clinic. Consented individuals had a non-polarized e-bandage covered by Tegaderm placed on their forearm. A MP enclosed in a biocompatible case was connected to the e-bandage and secured using skin tape. One day later, the device was removed and a follow-up phone call made six days later to assess for adverse effects.

**Results:**

Three volunteers wearing a non-polarized H_2_O_2_ e-bandage experienced no discomfort, irritation, allergic reaction, or skin discoloration after device removal and seven days post device placement.

**Conclusion:**

These preliminary studies combined with the previously performed murine bacterial biofilms work support further studies of this novel device for treating wound infections caused by antimicrobial resistant pathogens.

**Disclosures:**

**Robin Patel, MD**, a patent on Bordetella pertussis/parapertussis PCR issued, a patent on a device/method for sonication with royalties paid by Samsung to Mayo Clinic, a: See above|MicuRx Pharmaceuticals and BIOFIRE: Grant/Research Support|PhAST, Day Zero Diagnostics, Abbott Laboratories, Sysmex, DEEPULL DIAGNOSTICS, S.L., Netflix, Oxford Nanopore Technologies and CARB-X: Advisor/Consultant|Up-to-Date and the Infectious Diseases Board Review Course.: Honoraria

